# Grape Pomace as a Source of Phenolics for the Inhibition of Starch Digestion Enzymes: A Comparative Study and Standardization of the Efficacy

**DOI:** 10.3390/foods13244103

**Published:** 2024-12-18

**Authors:** Pedapati Siva Charan Sri Harsha, Vera Lavelli

**Affiliations:** Department of Food, Environmental and Nutritional Sciences, University of Milan, 20133 Milano, Italy; sriharshabio2006@gmail.com

**Keywords:** α-glucosidase, α-amylase, grape, phenolic, acarbose

## Abstract

The increase in the incidence of hyperglycemia and diabetes poses the challenge of finding cost-effective natural inhibitors of starch digestion enzymes. Among natural compounds, phenolics have been considered as promising candidates. The aims of this study were as follows: (a) to investigate the effectiveness of the inhibition of different winemaking byproducts towards intestinal brush border α-glucosidase and pancreatic α-amylase in vitro; (b) to calculate an efficacy index relative to the standard acarbose for the phenolic pool of winemaking byproducts, as well as for isolated phenolic compounds and for the phenolic pools of different plants studied in the literature, in order to rank winemaking byproducts with respect to the reference drug and other natural alternatives. Among winemaking byproducts, red grape skins showed the highest inhibitory activities towards both α-glucosidase and α-amylase, which were, on average, 4.9 and 2.6 µg acarbose equivalents/µg total phenolics (µg Ac eq/µg GAE), respectively. A correlation was observed between the total phenolic contents of red grape skins and their inhibitory effectiveness, which is useful for standardizing the efficacy of phenolic extracts obtained from different winemaking processes. In general, the inhibitory activity of the phenolic pool of grape skins was higher than those of isolated phenolic compounds, namely anthocyanins and monomeric and polymeric flavanols and flavonols, probably due to synergistic effects among compounds. Hence, bioactive phenolic fractions could be produced with the focus on functionality rather than purity, in line with the principles of sustainable processing. Based on the efficacy index developed to compare different phenolic compounds and phenolic-rich plants studied in the literature as starch digestion enzyme inhibitors, red grape skins proved to be cost-effective candidates.

## 1. Introduction

The ability of phenolics as inhibitors of starch digestion enzymes, such as α-amylase and α-glucosidase, has been the focus of different investigations, which have also been carefully recompiled by review studies [1]. In fact, the pharmacological approach to the treatment of type 2 diabetes consists of inhibiting starch digestion enzymes using synthetic drugs, especially acarbose; however, several side effects of this treatment have been described [2]. Hence, it would be advisable to find efficient natural inhibitors. Among the different potential sources of natural inhibitors, grape and grape-derived products have emerged as the most promising [3,4]. In particular, a large part of grape bioactive compounds, including anthocyanins, flavonols, and monomeric, dimeric, oligomeric, and polymeric flavanols, remains in grape pomace (skins and seeds) after the winemaking process, mainly due to their hydrophobicity and hydrogen bonds [3]. Red grape pomace (Cabernet Franc variety) extract was found to suppress postprandial hyperglycemia in diabetic mice following a potato starch challenge, and the effect was attributed to α-glucosidase inhibition [5]. In a further study, red grape skin extract (Norton variety) was found to significantly inhibit mammalian intestinal α-glucosidases in streptozocin-treated mice upon starch administration, but it was not effective on other digestive enzymes, including pancreatic α-amylase [6]. In a subsequent study, the intake of red grape pomace (Merlot variety) extract along with starch was assessed in rats. The positive effect observed was attributed to the inhibition of both pancreatic and salivary α-amylases, while no inhibition on α-glucosidase was detected [7]. A pilot study performed on eight human subjects showed that the postprandial plasma glucose concentrations after the ingestion of a high-carbohydrate meal with grape seed extract were significantly lower than those of the control subjects [8].

To obtain insights about the potential of grape phenolics as inhibitors of starch digestion enzymes, the effects of isolated compounds were assessed in vitro on α-glucosidase [9,10,11,12,13,14] and α-amylase [11,15,16,17,18,19,20,21,22]. Moreover, the combination of procyanidins with anthocyanins was found to improve the inhibition of starch digestion enzymes, likely due to synergistic effects [9]. Based on this latter finding, it is important to consider the raw phenolic extract as a pool, with a potentially higher effectiveness than the isolated components. This latter approach is also in line with the principle of sustainable food production, since food products/ingredients can become more sustainable if they are produced with the focus on functionality rather than purity [23]. Moreover, the possibilities to upgrade food byproducts is another principle of sustainable food production [23].

The existing literature on natural inhibitors of starch digestion enzymes has identified different natural sources of inhibitors, including grape pomace [1,3]. On the other hand, the efficacy of these sources was expressed with indexes that depend on the procedures applied in different laboratories, which were not standardized. As a result, no comparison among the proposed sources was possible [1,3].

Considering the existing information, there is a further gap to be filled to support the application of grape pomace phenolics to manage hyperglycemia. In fact, knowledge on the efficacy of the whole grape pomace phenolic pool, in relation to both the natural variability of this resource and the efficacy of other natural sources, is lacking. Hence, the aims of this manuscript were as follows: (a) to investigate the effectiveness of different grape pomace samples as inhibitors of intestinal brush border α-glucosidase and pancreatic α-amylase in vitro, and (b) to calculate an efficacy index relative to the standard acarbose for the grape pomace phenolic pool, different standard phenolic compounds, and phenolic-rich plants studied in the literature, in order to rank grape pomace phenolics with respect to other natural sources and the reference drug.

## 2. Materials and Methods

### 2.1. Chemicals

Standard phenolics, namely catechin, epicatechin, procyanidin B1, procyanidin B2, quercetin–3–O–glucoside, quercetin, kaempferol, delphinidin–3–O–glucoside, cyanidin–3–O–glucoside, petunidin–3–O–glucoside, peonidin–3–O–glucoside, malvidin–3–O-glucoside, gallic acid, rat intestinal acetone powder (N1377–5G), *p*-nitrophenyl α-D-glucoside (*p*-NPG), acarbose, porcine pancreatic α-amylase, type VI-B (A3176), *p*-nitrophenyl α-D-maltopentaoside (*p*-NPGP), methanol, ethanol, acetonitrile, hydrochloric acid, *n*-butanol, Folin–Ciocalteu reagent, sodium carbonate, ammonium iron(III) sulfate, phosphoric acid, and potassium phosphate, were obtained from Sigma Aldrich (Milan, Italy).

### 2.2. Grape Pomace

Pomace samples were collected from wineries located in Lombardia and Piemonte, which are Italian regions particularly devoted to winemaking. At the wineries, all of the processed grapes were from organic agriculture. A de-stemming step was applied, and the stems were discarded. Then, the grapes were crushed and the pomace (mixture of skins and seeds) was collected either before fermentation (for white grapes) or after fermentation (for red grapes). The collected pomace samples had no signs of mold attack. The samples included the red grape varieties Barbera (BA), Croatina (CR), Freisa (FR), Dolcetto (DO 01 and DO 02, corresponding to the same variety processed by two different wineries), Grignolino (GR), Neretto (NR), and Nebbiolo (NE) and the white grape varieties Arneis (AR), Moscato (MO), Muller-Thurgau (MT), Erbaluce (ER), Chardonnay (CH), Nascetta (NA), and Riesling (RI). A heterogeneous batch of red grape pomace was assembled by mixing the red pomace samples in equal amounts. The pomace samples were fractionated into seeds and skins using a 5 mm sieve and immediately frozen. The skins and seeds were dried at 50 °C for about 8 h using an Ignis model AKS201/IX/01 ventilated oven (Whirpool, Milan, Italy) [24]. The dried skins were then milled and sieved to obtain the fraction with particle sizes in the range of 250–500 µm (Octagon Digital sieve shaker, Endecotts Ltd., London, UK).

### 2.3. Phenolic Extraction

For sample comparison, about 100 mg of grape skin powder was extracted in duplicate with 8 mL of methanol/water/HCl (80:20:0.1, *v*/*v*/*v*), for 2 h at room temperature with continuous stirring. The mixture was centrifuged at 10,000× *g* for 10 min, the supernatant was recovered, and the solid residue was re-extracted using 6 mL of the same solvent twice. The three supernatants were pooled and dried under a vacuum at 35 °C, and the residue was suspended in 10 mL of methanol/water/HCl (80:20:0.1, *v*/*v*/*v*) for further analysis. Red grape skins separated from the heterogeneous pomace batch were extracted according to a food-grade protocol, by 60% aqueous ethanol with continuous stirring for 2 h at 60 °C [25].

### 2.4. Total Phenolics

The Folin–Ciocalteu assay was performed in duplicate as described previously and the total phenolic content was expressed as the milligrams of gallic acid equivalent (GAE) per gram of dry product or milligrams of GAE per liter of extract [24].

### 2.5. Procyanidins

The procyanidin content was analyzed in duplicate as described previously and expressed in milligrams per liter of extract, using 1.79 as a conversion factor [24].

### 2.6. Phenolic Characterization by HPLC

The phenolic contents were analyzed in duplicate as described previously [26], using a model Shimadzu LC−20 AD pump coupled to a model Shimadzu SPD-M20A photodiode array detector (DAD) and an RF−20 AXS operated by Labsolution Software 5.5 (Shimadzu, Kyoto, Japan). A 2.6 µm Kinetex C18 column (150 × 4.6 mm; Phenomenex, Bologna, Italy) was used for the separation at a flow rate of 1.5 mL/min. The column was maintained at 40 °C. The separation was performed by means of a linear gradient elution. The eluents were (A) 0.1% H_3_PO_4_ and (B) acetonitrile. The gradient was as follows: from 6% B to 20% B in 18 min; from 20% B to 60% B in 7 min; from 60% B to 90% B in 19 min; 90% B for 10 min; and then 6% B for 5 min. A DAD analysis was carried out in the range of 200–600 nm. The identification of compounds was achieved by comparing their retention times and UV-Vis spectra with those of authentic standards. The quantification of phenolics was performed by calibration curves built with cyanidin–3–O–glucoside for anthocyanins, with the DAD set at 520 nm; quercetin–3–O–glucoside for flavonols, with the DAD set at 354 nm; and catechin for monomeric and dimeric flavanols, with the fluorimetric detector set at λex 230 and λem 320. The results were expressed as the milligrams of phenolic compound per liter of extract.

### 2.7. In Vitro α-glucosidase and α-amylase Inhibition Assay

A crude α-glucosidase solution was prepared by dissolving 200 mg of rat intestinal acetone powder in 4 mL of 50 mM ice-cold phosphate buffer (pH of 6.8) and sonicating for 15 min at 4 °C. The resulting suspension was vortexed for 20 min and then centrifuged at 10,000× *g* at 4 °C for 30 min, and the supernatant was used for the assay. For the determination of α-glucosidase activity, 650 µL of 50 mM phosphate buffer, with a pH of 6.8; 100 µL of the enzyme solution; and 50 µL of grape skin extract were added to an Eppendorf tube and pre-incubated for 5 min at 37 °C. Then, 200 µL of 1 mM *p*NPG was added as the substrate and the mixture was further incubated at 37 °C for 25 min. For the determination of pancreatic α-amylase activity, 550 µL of 50 mM phosphate buffer, with a pH of 6.8; 200 µL of the enzyme solution (10 μM in the same buffer); and 50 µL of grape skin extract were added to an Eppendorf tube and pre-incubated for 5 min at 37 °C. Then, 200 µL of 1 mM *p*NPGP was added to the tube as the substrate and the mixture was further incubated at 37 °C for 55 min. For both enzymatic reactions, the assay mixture was centrifuged at 10,000× *g* for 3 min and the absorbance of the clear supernatant was recorded at 405 nm. The control was run by adding the extraction solvent, replacing the sample in order to nullify the possible interference of the solvent on the enzymatic activity. A sample blank and a control blank were run without the addition of a substrate and without the addition of either the substrate or the sample, respectively [27]. Acarbose was used as a reference inhibitor for both enzymatic reactions. Five-point dose–response curves were made in duplicate for the samples and for the reference compound.

### 2.8. Efficacy of Inhibition

To study the efficacy of enzyme inhibition relative to the mass unit of grape skins, the I50 sample, i.e., the concentration that inhibited the reaction by 50%, was interpolated from the dose–response curve of every sample and expressed as the milligrams of grape skins per milliliter of assay solution. I50 is commonly used as an index to evaluate enzyme inhibition, but it depends on many factors such as the enzyme and substrate concentrations, as well as the assay conditions (pH, temperature, and type of buffers used). Hence, from the dose–response curve of acarbose, the I50 acarbose was calculated and expressed as the micrograms of acarbose per milliliter of assay mixture. Then, the efficacy of the inhibition of grape skins relative to acarbose was calculated as the microgram of acarbose equivalents per milligram of grape skins, as follows:

µg Ac eq/mg sample = I50 acarbose/I50 sample(1)

To study the inhibitory properties of the phenolic pool of grape skins, the efficacy of inhibition was also expressed as the microgram of acarbose equivalents per microgram of phenolics (GAE), as follows:

µg Ac eq/µg GAE = (µg Ac eq/mg sample)/(µg GAE/mg sample)(2)

For comparing the inhibitory properties of the phenolic pool of grape skins with those of phenolic-rich plants reported in the literature, only the studies that used acarbose as a reference compound were considered. Then, the efficacy of the inhibition of the plant sample was re-calculated using Equation (2).

The efficacy of standard phenolic compounds reported in literature studies that used acarbose as a reference compound was also re-calculated as the ratio between the I50 of acarbose in micrograms per milliliter of assay mixture and their I50 values expressed as the micrograms per milliliter of assay mixture, as follows:

µg Ac eq/µg phenol = I50 acarbose/I50 phenolic compound(3)

### 2.9. Statistical Analysis of Data

The experimental data were analyzed by one-way ANOVA using the least significant difference (LSD) as a multiple range test, and by non-linear regression analyses using Statgraphics 5.1 (STCC Inc.; Rockville, MD, USA). The results are reported as the average ± the standard deviation (SD).

## 3. Results

### 3.1. α-glucosidase and α-amylase Inhibition

**Figure 1:**
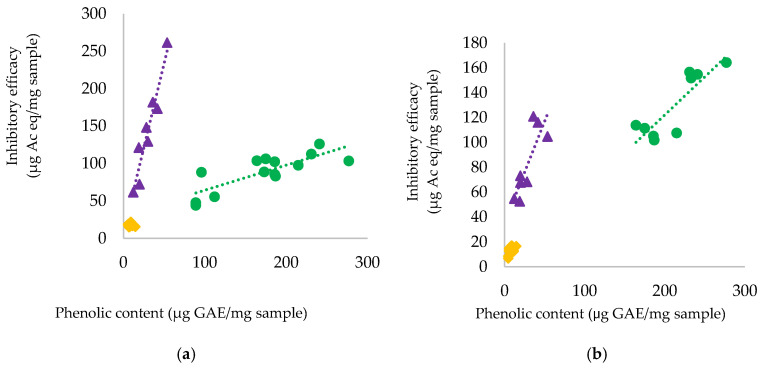
Correlation between enzyme inhibition efficacy and phenolic content for red grape skins (▲), white grape skins (♦), and grape seeds (●). (**a**) α-glucosidase inhibition; (**b**) α-amylase inhibition. Data for grape seeds were recalculated from Lavelli et al., 2015 [27].

Grape skin phenolics were extracted from eight red grape skin and seven white grape skin batches recovered from winemaking byproducts, and the inhibitory activity of the extracts toward starch digestion enzymes was assessed (Table 1). The correlation between the total phenolic content of the extracts and the starch digestion enzyme inhibition was then investigated (Figure 1a,b).

#### 3.1.1. α-glucosidase Inhibition

The I50 values for α-glucosidase inhibition ranged between 0.38 and 1.66 mg skins/mL, corresponding to 261–62 µg Ac eq/mg skins for red grape skins and between 4.62 and 6.45 mg skins/mL, corresponding to 21.6 and 15.5 µg Ac eq/mg skins (Table 1). A previous study has shown that fourteen batches of grape seeds had I50 values ranging from 0.80 to 2.37 mg seeds/mL, corresponding to 126–44 µg Ac eq/mg seeds [27].

Considering red grape skins, the inhibition effectiveness toward α-glucosidase was correlated with the procyanidin content (R = 0.922, *p* < 0.05) and anthocyanin content (R = 0.893, *p* < 0.05), while no correlation was found with the flavonol content. However, the best correlation was observed between α-glucosidase and the total phenolic content (R = 0.966, *p* < 0.05, Figure 1a). The inhibitory activity of red grape skins calculated with respect to the phenolic content ranged between 3.8 and 5.3 µg Ac eq/µg GAE, and the slope of the correlation curve was 4.9 ± 0.5 µg Ac eq/µg GAE.

Among white grape skins, the inhibitory activity toward α-glucosidase was similar, and it was lower than that for red grape skins. No significant correlation was observed between the inhibitory activity and the contents of procyanidins, flavonols, and total phenolics. The inhibitory activity of red grape skins, calculated with respect to the phenolic content, ranged between 1.0 and 3.8 µg Ac eq/µg GAE. As shown previously, the inhibitory activity of grape seeds toward α-glucosidase was correlated with the total phenolic content (R = 0.8033, *p* < 0.05), with an average value of 0.33 µg Ac eq/µg GAE [27]. Hence, the phenolic pool of red grape skins was 10-fold more efficient than that of grape seeds.

#### 3.1.2. α-amylase Inhibition

Considering α-amylase inhibition, the I50 values of red grape skins ranged between 0.21 and 0.57 mg skins/mL, corresponding to 145–52 µg Ac eq/mg skins. For white grape skins, the I50 values ranged between 1.77 and 2.44 mg skins/mL, corresponding to 16.9–12.4 µg Ac eq/mg skins (Table 1). In a previous study, fourteen batches of grape seeds were found to have I50 values ranging from 0.19 to 0.31 mg seeds/mL, corresponding to 164–98 µg Ac eq/mg seeds [27].

For red grape skins, a significant correlation was observed between the inhibition effectiveness toward α-amylase and the total phenolic content (R = 0.836, *p* < 0.10, Figure 1b), as well as the anthocyanin content (R = 0.760, *p* < 0.10) and procyanidin content (R = 0.755, *p* < 0.10), but no correlation was observed with the flavonol content. Based on the phenolic content, the inhibitory effectiveness was in the range of 2.3–3.6 µg Ac eq/µg GAE. From the slope of the correlation curve between the enzyme inhibition and the total phenolic content, it can be derived that red grape skins can provide an average of 2.6 ± 0.7 µg Ac eq/µg GAE. The inhibitory activity of white grape skins varied in the range of 1.1–1.9 µg Ac eq/µg GAE.

The phenolic pool of red grape skins was generally a more efficient inhibitor than that of grape seeds, which had 0.61 µg Ac eq/µg GAE [27]. However, grape seeds generally had a higher phenolic content than grape skins, and hence, they resulted in more acarbose equivalents per mass unit.

### 3.2. Characterization of Food-Grade Extracts of Red Grape Skins

**Figure 2:**
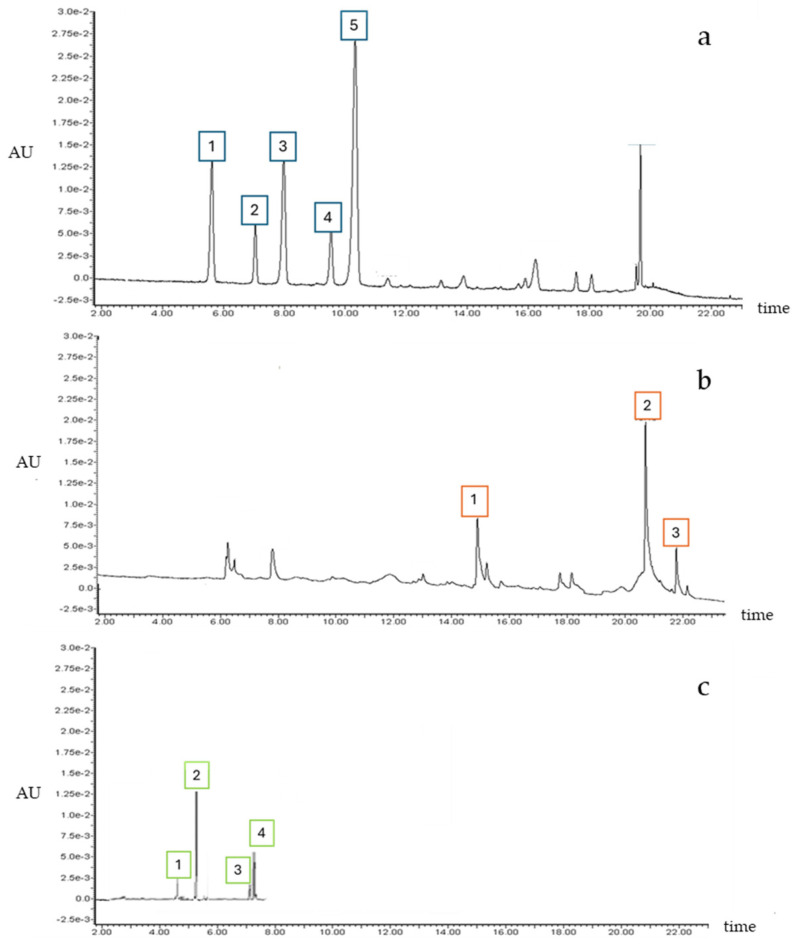
HPLC chromatograms of red skin extract. (**a**) Major peaks identified at 520 nm: 1. delphinidin–3–O–glucoside; 2. cyanidin–3–O–glucoside; 3. petunidin 3–O–glucoside; 4. peonidin–3–O–glucoside; and 5. malvidin–3-O–glucoside. (**b**) Major peaks identified at 354 nm: 1. quercetin glucoside; 2. quercetin; and 3. kaempferol. (**c**) Major peaks identified at λex 230/λem 320: 1. procyanidin B1; 2. catechin; 3. procyanidin B2; and 4. epicatechin.

The phenolic fraction of a heterogeneous batch (mixture of varieties) of red grape skins was then recovered by a procedure that yields a food-grade extract with a good stability, showing its potential to be considered for food applications [24]. In fact, since grape pomace is a byproduct, it is generally collected as a whole, without any separation according to the grape varieties or winemaking process. As expected, the phenolic profile included anthocyanins, flavonols, and monomeric, dimeric, and procyanidins (Figure 2, Table 2).

The inhibitory activity towards starch digestion enzymes was then determined and found to be 5.2 and 2.0 µg Ac eq/µg GAE for α-glucosidase and α-amylase, respectively (Table 3 and Table 4). These values are close to those expected from the correlations between the total phenolic content and the inhibitory efficacy for α-glucosidase (4.9 ± 0.5 µg Ac eq/µg GAE) and α-amylase (2.6 ± 0.7 µg Ac eq/µg GAE), suggesting that the correlation found could be used to predict the enzyme inhibition effectiveness based on the phenolic content, which can vary depending on genetic factors, the climate, cultivation practices, and winemaking procedures such as the use of spontaneous or inoculated fermentation [28].

## 4. Discussion

Previous studies have found that natural compounds have higher inhibitory activity towards yeast α-glucosidase than rat intestinal α-glucosidase [29,30]. Hence, the inhibitory activity of winemaking byproducts was compared to those of standard compounds and plant extracts assessed using rat intestinal α-glucosidase. Moreover, the comparison was made only when acarbose was used as a positive control and the activity could be measured as acarbose equivalents, in order to minimize possible differences due to the assay conditions (Table 3).

The main grape anthocyanin, i.e., malvidin–3–O–glucoside, was found to be a more effective inhibitor than acarbose (3.99 µg Ac eq/µg) [10]; similarly, the other grape anthocyanins were more efficient than acarbose [10], except for cyanidin–3–O–glucoside [9,10,11]. Flavonols were generally 10-fold less efficient than acarbose [12,14], except for quercetin glucoside [14]. Catechin, epicatechin, and procyanidin B1 were found to be 20 times less efficient than acarbose [13]. For cyanidin–3–O–glucoside, quercetin, and kaempferol –3–O–glucoside, the observed effectiveness was not the same for the different studies. This result could be due to the different assay conditions used. Anthocyanins induce a mixed type of inhibition against α-glucosidase. CD studies have shown that anthocyanin binding to this enzyme decreases the percentage of α-helixes and increases the percentage of β-sheets, indicating that the secondary structure of the enzyme is altered [10]. Moreover, molecular docking studies have shown that anthocyanins can enter in depth into the active pocket of α-glucosidase and form hydrophobic interactions with Glu276, Asp214, Ala278, Tyr71, and Asp349 residues; in addition, their glycosyl groups can interact with Asp408 via hydrogen bonds [10]. Overall, the phenolic pool of red grape skins observed in the current study was found to be more efficient than the purified compounds, confirming the importance of the synergistic effects among antioxidants, as previously observed [9].

The inhibitory activity towards rat intestinal α-glucosidase was also investigated for different berries (Table 3), including blackcurrants, rowanberries, raspberries, cloudberries, and blackberries [9,11]. In all these fruits, the inhibitory activity was lower than that found for red grape skins. Red cabbage was found to be an efficient source of rat intestinal α-glucosidase inhibitors, comparable to that of red grape skins, with a variety-dependent effect [31]. The high inhibitory activity of red cabbage was attributed to the content of anthocyanins, especially the diacylated forms, which could act synergistically with hydroxycinnamic acids [30]. Among potential rat intestinal α-glucosidase inhibitors, roasted ground coffee was one anthocyanin-free source. It was less efficient than red grape skins, and its activity was tentatively attributed not only to phenolics, but also to the Amadori compounds and melanoidins [32]. Another anthocyanin-free source that is receiving attention is the fruiting body of mushrooms [30]. Indeed, an inhibitory activity comparable to that found in grape skins was observed in mushrooms, which was attributed to pyrrole alkaloids [30].

**Table 3:** Effectiveness of standard phenolics, red grape skin extract, and different plant extracts as rat intestinal α-glucosidase inhibitors.

Compound/Plant	Solvent	µg Ac eq/µg Phenolic or µg Ac eq/µg GAE	Ref.
catechin		<0.047	[13]
epicatechin		<0.047	[13]
procyanidin B1		<0.047	[13]
malvidin 3–O–glucoside		3.99	[10]
cyanidin–3–-O–glucoside		0.26, 0.46, 0.52	[9,10,11]
delphinidin–3–O–glucoside		3.46	[10]
petunidin–3–O–glucoside		3.12	[10]
quercetin		0.22, 0.09	[12,13]
quercetin–3–O–glucoside		0.28	[14]
kaempferol		0.093	[13]
kaempferol–3–O–glucoside		0.12, 0.16	[13,14]
blackcurrants (*Ribes nigrum*)	fruits, 0.2% formic acid in water	2.0	[9]
rowanberries (*Sorbus aucuparia*)	fruits, 0.2% formic acid in water	1.3	[9]
raspberries (*Rubus idaeus*)	fruits, 0.2% formic acid in water	<0.2	[9]
cloudberries (*Rubus chamaemorus*)	fruits, 0.2% formic acid in water	<0.2	[9]
blackberries (*Rubus grandifolius*)	fruits, 80% methanol, 7% acetic acid	0.4	[11]
blackberries (*Rubus grandifolius*)	fruits, 80% methanol, 7% acetic acid	0.4	[11]
blackberries (*Rubus grandifolius*)	leaves, 80% methanol, 7% acetic acid	0.5	[11]
blackberries (*Rubus grandifolius*)	leaves, 80% methanol, 7% acetic acid	0.6	[11]
red cabbage (*Brassica oleracea*)	edible part, 70% methanol	5.8	[31]
red cabbage (*Brassica oleracea*)	edible part, 70% methanol	8.3	[31]
red cabbage (*Brassica oleracea*)	edible part, 70% methanol	5.4	[31]
red cabbage (*Brassica oleracea*)	edible part, 70% methanol	6.6	[31]
red cabbage (*Brassica oleracea*)	edible part, 70% methanol	4.3	[31]
coffee (*Coffea arabica*)	ground roasted beans, water	1.7	[32]
mushrooms (*Grifola frondose*)	fruiting body, 95% ethanol	4.5	[30]
grapes (*Vitis vinifera*)	red skins, 60% ethanol	5.2 ± 0.1	this study

Regarding α-amylase inhibition by standard compounds and plant extracts, only studies in which porcine α-amylase was used and acarbose was included as a positive control were considered (Table 4). Very low inhibitory activity was observed for anthocyanins [11,17,18], flavonols [19], and monomeric flavanols [15,16,17]. In one study, red grape phenolics were fractionated and the efficacy of the procyanidin fraction was found to be higher (1.19 µg Ac/µg) with respect to that of the anthocyanin fraction (0.74 µg Ac eq/µg) [21]. For procyanidins, besides their total amount, their structural features are also relevant for the inhibition of enzymatic activity. Indeed, a purified fraction of grape seed procyanidins was found to be more effective than acarbose (6.1 µg Ac eq/µg), while the same fraction lost effectiveness upon depolymerization with mercaptoethanol [20]. In a further study, a lower effectiveness was observed for grape seed procyanidins with respect to acarbose (0.793 µg Ac eq/µg) [16], probably due to the effect of the grape varieties and/or the procedures applied for purification on the molecular weight of the fractionated compounds. Indeed, the procyanidin fraction with a mean polymerization degree of 1.35 showed 0.28 µg Ac eq/µg, while that with a mean polymerization degree of 6.97 showed 6.77 µg Ac eq/µg [22]. Procyanidins have a strong hydrogen-bonding capacity, and hence, they can interact with different proteins [20]. Hence, these molecules were supposed to inhibit enzyme activity through non-specific binding by preventing interactions with substrates [9]. Indeed, procyanidins were found to be able to precipitate digestive enzymes during simulated digestion [33]. However, molecular docking studies have demonstrated that grape procyanidins can specifically interact with the active site of α-amylase through hydrogen interactions and hydrophobic and electrostatic interactions, which are stronger for procyanidin tetramers than for the trimers or the dimers [22]. In fact, in the catalytic core of α-amylase, seven hydrogen bonds can be formed between the procyanidin tetramers and the enzyme.

Overall, the phenolic pool of red grape skins was more efficient than acarbose as an inhibitor of α-amylase. However, a higher efficacy could be obtained by the purification of the procyanidin faction. Various plants considered as sources of α-amylase inhibitors, such as rowanberries, hibiscus, and dogwoods, displayed a lower effectiveness with respect to red grape skins, while coffee and saffron displayed a comparable effectiveness [9,32,33,34,35,36] (Table 4). On the other hand, elderberries were found to be more effective than red grape skins, most probably due their procyanidin structure [37]. Moreover, red sweet peppers have been highlighted as possible potent α-amylase inhibitors, more efficient than red grape skins, due to their carvacrol content [38].

**Table 4:** Effectiveness of standard phenolics, red grape skin extract, and different plant extracts as porcine pancreatic α-amylase inhibitors.

Compound/Plant	Solvent	µg Ac eq/µg Phenolic or µg Ac eq/µg GAE	Ref.
catechin		0.0021, 0.043	[15,16]
epicatechin		Nd	[15,16,17]
cyanidin–3–O–glucoside		0.020, 0.044	[11,18]
quercetin		<0.027	[19]
kaempferol		<0.028	[19]
PA, mDP not specified		6.1, 1.2	[20,21]
PA, mDP: 1.35%, G: 5.43		0.28	[22]
PA, mDP: 6.97%, G: 13.27		6.77	[22]
rowanberries (*Sorbus aucuparia*)	fruits, 0.2% formic acid in water	0.2	[9]
hibiscus (*Hibiscus deflersii*)	aerial part, water	0.6	[34]
hibiscus (*Hibiscus calyphyllus*)	aerial part, water	0.3	[34]
hibiscus (*Hibiscus micranthus*)	aerial part, water	0.2	[34]
dogwoods (*Cornus mas*)	fruits, 60% ethanol extract	0.8	[35]
dogwoods (*Cornus alba*)	fruits, 60% ethanol extract	0.7	[35]
elderberries (*Sambucus nigra*)	edible part, 50% ethanol	8.34	[37]
saffron (*Crocus sativus*)	stigma, water extract	1.98	[36]
saffron (*Crocus sativus*)	tepal, water extract	2.32	[35]
coffee (*Coffea arabica*)	ground roasted beans, water	1.49	[32]
red peppers (*Capsicum annuum*)	edible part, 70% ethanol	8.42	[38]
grapes (*Vitis vinifera*)	red skins, 60% ethanol	2.0 ± 0.1	this study

PA, procyanidins; mDP, mean polymerization degree; G, galloyl groups.

The search for new drugs that can act as inhibitors of α-glucosidase and α-amylase is intensive [39,40]. Based on the results obtained for grape pomace samples and on the comparison among different plants and the reference drug acarbose, red grape skins can represent a target source for the drug industry to develop drug alternatives.

## 5. Conclusions

To sum up, this study led to the following conclusions:

(a) Among winemaking byproducts, red grape skins showed the most efficient ability to inhibit starch digestion enzymes, which was correlated with the total phenolic content (GAE). The observed correlation provides a useful tool to facilitate the scaling-up of this application.
(b) The efficacy of the inhibition by red grape skins was higher than that of isolated phenolics, probably due to synergistic effects among compounds.
(c) Based on the standardized efficacy index developed in this study, the inhibitory activity of red grape skins was found to be generally higher than those of other plants considered in the literature, especially towards α-glucosidase.

The comparison among various plant extracts considered in the literature as sources of starch digestion enzyme inhibitors was limited because some studies lacked the specification of the enzyme source used in the assay; used yeast α-glucosidase instead of rat intestinal α-glucosidase; and/or did not run an internal control (acarbose). Moreover, for natural sources, biodiversity cannot be ruled out, and hence, different varieties of the same plant source should be analyzed to assess the inhibitory efficacy.

Despite these limitations, the role of red grape skins as a potential source of inhibitors of starch digestion enzymes is promising. Additionally, the use of unpurified fractions instead of isolated compounds and the opportunity to upgrade food byproducts are in line with the principles of sustainable food processing.

## Figures and Tables

**Table 1:** Efficacy of inhibition of rat intestinal α-glucosidase and porcine pancreatic α-amylase by phenolic extracts of grape skins recovered from winemaking byproducts ^1,2^.

Sample	α-glucosidase Inhibition		α-amylase Inhibition	
	I50, mg Sample/mL	µg Ac eq/mg Sample	I50, mg Sample/mL	µg Ac eq/mg Sample
Red Skins				
DO 01	0.77 ^ab^ ± 0.03	130 ^cd^ ± 7	0.24 ^a^ ± 0.02	118 ^d^ ± 3.9
BA	0.68 ^ab^ ± 0.04	148 ^de^ ± 12	0.44 ^ab^ ± 0.01	68.3 ^c^ ± 5.3
FR	0.38 ^a^ ± 0.02	261 ^f^ ± 21	0.21 ^a^ ± 0.00	145 ^e^ ± 0.9
CR	0.58 ^ab^ ± 0.03	173 ^e^ ± 13	0.21 ^a^ ± 0.03	145 ^e^ ± 3.2
NE	1.39 ^b^ ± 0.29	72 ^c^ ± 8	0.42 ^ab^ ± 0.02	70 ^c^ ± 3.5
NR	1.66 ^c^ ± 0.26	62 ^b^ ± 13	0.55 ^b^ ± 0.02	55 ^b^ ± 1.9
GR	0.83 ^ab^ ± 0.05	121 ^cd^ ± 10	0.57 ^b^ ± 0.00	52 ^b^ ± 1.5
DO 02	0.55 ^ab^ ± 0.03	181 ^e^ ± 10	0.25 ^a^ ± 0.01	118 ^d^ ± 2.3
White Skins				
MO	5.51 ^ef^ ± 0.28	18.1 ^a^ ± 0.1	2.23 ^d^ ± 0.18	14.0 ^a^ ± 1.1
AR	4.62 ^d^ ± 0.08	21.6 ^a^ ± 0.4	1.77 ^c^ ± 0.10	16.9 ^a^ ± 0.1
ER	5.36 ^e^ ± 0.54	18.8 ^a^ ± 0.1	1.85 ^c^ ± 0.11	16.2 ^a^ ± 0.9
MT	6.42 ^g^ ± 0.10	15.6 ^a^ ± 0.1	1.82 ^c^ ± 0.01	16.5 ^a^ ± 0.1
CH	5.88 ^fg^ ± 0.44	16.7 ^a^ ± 0.6	2.26 ^d^ ± 0.24	13.1 ^a^ ± 1.4
NA	6.45 ^g^ ± 0.29	15.5 ^a^ ± 0.7	2.44 ^d^ ± 0.30	12.4 ^a^ ± 1.5
RI	5.69 ^efg^ ± 0.20	17.1 ^a^ ± 0.6	2.38 ^d^ ± 0.12	12.6 ^a^ ± 0.6

^1^ Values are the means ± SD. Different lowercase letters in the same column (a–g) indicate significant differences (LSD, *p* < 0.05). ^2^ The I50 values for acarbose were 100 and 30 µg/mL for α-glucosidase and α-amylase inhibition, respectively.

**Table 2:** Phenolic contents of the 60% ethanol extract of a heterogeneous batch of red grape skins.

Parameter	mg/mL
delphinidin–3–O–glucoside	34.5 ± 1.4
cyanidin–3–O–glucoside	14.9 ± 0.6
petunidin–3–O–glucoside	44.4 ± 1.8
peonidin–3–O–glucoside	17.9 ± 0.7
malvidin–3–O–glucoside	93.4 ± 3.7
quercetin–3–O–glucoside	29.7 ± 1.2
quercetin	27.2 ± 1.1
kaempferol	5.2 ± 0.2
procyanidin B1	6.6 ± 0.3
catechin	37.3 ± 1.5
procyanidin B2	10.3 ± 0.4
epicatechin	17.5 ± 0.7
procyanidins	3180 ± 130
total phenolics (GAE)	5490 ± 220

## Data Availability

The original contributions presented in this study are included in the article; further inquiries can be directed to the corresponding author.
